# An Application for Urticaria

**Published:** 1898-02

**Authors:** 


**An Application for Urticaria.**—Gaucher (cited in the *Gazette Hebdomadaire de Medicine et de Chirurgie* for July) recommends this:

℞. Mentholis..........................................1 part,
Chloroformi,
Etheris,
Spts. camphoris..........................aa 3 parts.

M. To be used as a spray or as a lotion. The part should then be dusted with powdered starch or zinc oxide.—*Medical Review of Reviews.*

